# Variance component analysis of growth and production traits in Vanaraja male line chickens using animal model

**DOI:** 10.5713/ajas.19.0826

**Published:** 2020-04-12

**Authors:** Rajkumar Ullengala, L. Leslie Leo Prince, Chandan Paswan, Santosh Haunshi, Rudranath Chatterjee

**Affiliations:** 1 ICAR-Directorate of Poultry Research, Rajendranagar, Hyderabad 500030, Telangana, India

**Keywords:** Animal Model, Restricted Maximum Likelihood (REML), Variance, Genetic Parameters, Economic Traits

## Abstract

**Objective:**

A comprehensive study was conducted to study the effects of partition of variance on accuracy of genetic parameters and genetic trends of economic traits in Vanaraja male line/project directorate-1 (PD-1) chicken.

**Methods:**

Variance component analysis utilizing restricted maximum likelihood animal model was carried out with five generations data to delineate the population status, direct additive, maternal genetic, permanent environmental effects, besides genetic trends and performance of economic traits in PD-1 chickens. Genetic trend was estimated by regression of the estimated average breeding values (BV) on generations.

**Results:**

The body weight (BW) and shank length (SL) varied significantly (p≤0.01) among the generations, hatches and sexes. The least squares mean of SL at six weeks, the primary trait was 77.44±0.05 mm. All the production traits, viz., BWs, age at sexual maturity, egg production (EP) and egg weight were significantly influenced by generation. Model four with additive, maternal permanent environmental and residual effects was the best model for juvenile growth traits, except for zero-day BW. The heritability estimates for BW and SL at six weeks (SL6) were 0.20±0.03 and 0.17±0.03, respectively. The BV of SL6 in the population increased linearly from 0.03 to 3.62 mm due to selection. Genetic trend was significant (p≤0.05) for SL6, BW6, and production traits. The average genetic gain of EP40 for each generation was significant (p≤0.05) with an average increase of 0.38 eggs per generation. The average inbreeding coefficient was 0.02 in PD-1 line.

**Conclusion:**

The population was in ideal condition with negligible inbreeding and the selection was quite effective with significant genetic gains in each generation for primary trait of selection. The animal model minimized the over-estimation of genetic parameters and improved the accuracy of the BV, thus enabling the breeder to select the suitable breeding strategy for genetic improvement.

## INTRODUCTION

Diversity in the populations exists due to the variability in genetics, environment and their interaction effects which form the basis for any genetic improvement program. Selection experiments continue to be powerful tools to generate information on quantitative traits in terms of their underlying genetic variability, the relationships between traits and their effects on performance [1]. Genetic progress in the population is determined by the response to selection for primary as well as other traits of economic importance [2]. The selection response depends on the estimated genetic and environmental parameters of different economic traits. Therefore, thorough understanding of genetic basis and action of different gene effects on economic traits along with the role of environment is essential for planning breeding strategies for obtaining improvement in the economic traits. The magnitude and direction of direct response to primary trait and correlated responses to other economic traits are significant in developing effective breeding strategies for improved productivity, more so in case of positively correlated traits due to linkage and pleiotropy.

Precise estimation of genetic parameters (heritability and correlation) plays a major role in determining the realized response in the economic traits. For precise estimates, the variance needs to be partitioned and attributed to maximum possible sources and to their interaction effects that lead to reduced error variance and minimized over weightage to some components. Animal model is a powerful tool for the geneticist to accommodate additional components of variance, which makes the estimates less biased by confounding environmental variation and explicit modeling of environmental (co) variance [3].

Determining the breeding value (BV) with the objective to select the parents for next generation is very important for the success of any breeding program. The precise estimation of BV depends on the effects considered in the statistical model in most of the cases. Additive genetic variance primarily determines the heritability of the trait, although non-genetic components are also important factors in determining the variability of the traits. Maternal effects play a significant role in development and expression of the economic traits due to genetic or environmental differences between dams or by the combination of genetic or environmental differences [4,5]. The inclusion of maternal effects in the model reduces the bias in genetic parameter estimation and also increases the precision of the estimates [6]. Recent studies revealed that the hatch weight of the chicks is greatly influenced by the maternal additive genetic effects and is positively associated with egg quality traits [7]. Many authors confined their estimates to the direct estimation of genetic parameters without considering the maternal effects in chicken [8–13]. Some publications with additive, maternal and permanent effects on various economic traits using diallel analysis were reported in chicken [8,14]. However, limited studies are available with vast data and robust animal model analysis from India.

Analysis of vast data from more number of generations using robust algorithm like restricted maximum likelihood (REML) will delineate the status and structure of the population with respect to genetic variability, genetic trends, inbreeding etc. Therefore, the present study was carried out with an aim to delineate the population status and direct additive, maternal genetic and permanent environmental effects, besides genetic trends and performance with respect to growth and production traits in Vanaraja male line/project directorate -1 (PD-1) chicken.

## MATERIALS AND METHODS

The study was carried out at the experimental poultry farm of Indian Council of Agriculture Research (ICAR)-Directorate of Poultry Research, Hyderabad, India. Hyderabad is located in Deccan plateau in southern part of India positioned between 17°23’ N and 78°28’ E at height of 500 m from sea level. The region experiences tropical environment with hot summer (33°C to 45°C) and pleasant winter (16°C to 20°C) conditions. The poultry houses were covered with paddy straw with sprinklers to reduce the shed temperature in hot summer (March to June). The experiment was approved by the Institutional Animal Ethics Committee vide approval No. IAEC/DPR/17/4.

### Experimental population and management

Vanaraja male line (PD-1) was derived from a low performing Red Cornish population which has been under selection for higher 6 week shank length (SL6) since last eight generations from 2010–11. Higher SL is one of the major selection criterion in rural poultry lines as it enable the birds to move faster in free range conditions and reduces the predation. PD-1 is the male parent line for production of *Vanaraja*, a popular dual purpose rural chicken variety developed by ICAR-Directorate of Poultry Research.

Chicks were hatched in 3 to 4 hatches in each generation in a pedigreed mating with 50 sires and 250 dams, each sire mated to five dams (1:5). In each generation, about 3,000 healthy chicks were produced; wing banded and reared on deep litter in an open sided poultry house. Standard brooding, feeding and management practices were followed. The chicks were fed broiler starter ration with 2,900 kcal metabolisable energy (ME) and 22.0% crude protein (CP) *ad-libitum* up to six weeks of age. The chicks were vaccinated against Marek’s disease (MD), Newcastle disease (ND), infectious bursal disease (IBD) and fowl pox on zero, 5th, 14th, and 21st day, respectively. Body weight (BW) data were recorded at zero day, two, four, and six weeks of age, while SL was measured at six weeks of age. At the end of the six weeks, 450 females and 200 males were selected in each generation based on higher SL6, the primary trait of selection. The birds were kept on feed restriction schedule from seventh week onwards to maintain the target BW at laying. The birds were fed with broiler grower (2,850 kcal ME and 18.0% CP) ration till 16 weeks of age and broiler breeder (2,650 kcal ME and 16.5% CP) ration from 17 weeks to end of the production cycle (72 weeks). The adult birds were vaccinated against ND and IBD at 22 weeks of age and Infectious Bronchitis at 25 weeks of age.

### Data and traits studied

The data on growth and production performance of PD-1 line chickens collected over five generations (S7 to S11) from 2013–14 to 2018–19 were utilized in the present study, which were numbered serially from 1 to 5. In each generation, juvenile BW at zero day (BW0), two (BW2), four (BW4), and six (BW6) weeks; SL6; adult BWs at 20 (BW20) and 40 (BW40) weeks of age were measured. The BWs were measured to 0.1 g accuracy using digital balance while SL was measured to the nearest of 0.01 mm accuracy using digital Vernier calipers. Age at sexual maturity (ASM), part period egg production (EP) up to 40 weeks of age (EP40) and egg weight (EW) at 40 weeks (EW40) were recorded. The weight of eggs was recorded using a digital balance to an accuracy of 0.01 g. The detailed characteristics of data are presented in Table 1.

### Statistical analysis

Variance and covariance components were estimated by REML fitting an animal model [15]. Data were first analyzed by least squares analysis of variance (SPSS 12) to identify the fixed effects to be included in the model. Two statistical models were used for identifying the significant effects in the traits. For BW0, BW2, BW4, ASM, BW20, BW40, EP40, and EW40, the statistical model included the fixed effect of generation (five levels) and hatch number (four levels). For BW6 and SL6, the model included the effect of sex (two levels) in addition to generation and hatch effects. For juvenile and production traits, the generation effect was significant. Hatch effect was significant for all the juvenile growth traits and ASM. Sex of the chick significantly affected the BW6 and SL6. Only significant effects (p≤0.05) were included in the models which were subsequently used for the genetic analysis. Convergence of the REML solutions was assumed when the variance of function values (−2log L) in the simplex was less than 10^−8^. To ensure that a global maximum is reached, analyses were restarted and continued till convergence. Univariate animal models were fitted to estimate (co)variance components for all the traits. Different models which account for the direct and maternal effects were constructed as follows:

(1) $$y=X\beta +Z_{a}a+ɛ$$

(2) $$y=X\beta +Z_{a}a+Z_{m}m+ɛ\text{withCov }({\text{a}}_{\text{m}},{\text{m}}_{\text{o}})=0$$

(3) $$y=X\beta +Z_{a}a+Z_{m}m+ɛ\text{withCov }({\text{a}}_{\text{m}},{\text{m}}_{\text{o}})=A\sigma_{\text{am}}$$

(4) $$y=X\beta +Z_{a}a+Z_{\mathrm{pe}}\mathrm{pe}+ɛ$$

(5) $$y=X\beta +Z_{a}a+Z_{m}m+Z_{\mathrm{pe}}\mathrm{pe}+ɛ\text{withCov }({\text{a}}_{\text{m}},{\text{m}}_{\text{o}})=0$$

(6) $$y=X\beta +Z_{a}a+Z_{m}m+Z_{\mathrm{pe}}\mathrm{pe}+ɛ\text{withCov }({\text{a}}_{\text{m}},{\text{m}}_{\text{o}})=A\sigma_{\text{am}}$$

Where **y** is the vector of records; ***β***, **a**, **m**, **pe**, and ***ɛ*** are vectors of fixed, direct additive genetic, maternal additive genetic and permanent environmental effects of the dam, and residual effects, respectively; with association matrices **X**, **Z_a_**, **Z_m_**, and **Zpe**; **A** is the numerator relationship matrix between animals; and σ_am_ is the covariance between additive direct and maternal genetic effects. Assumptions for variance (**V**) and covariance (**Cov**) matrices involving random effects were

$$V(\text{a})=A{\sigma^{2}}_{\text{a}},V(\text{m})=A{\sigma^{2}}_{\text{m}},V(\text{c})=I{\sigma^{2}}_{\text{c}},V(\text{e})=I{\sigma^{2}}_{\text{e}},\;\text{and }\mathrm{Cov}(\text{a},\text{m})=A\sigma_{\text{am}}$$

Where, I is an identity matrix and σ ^2^_a_, σ^2^_m_, σ^2^_c_, and σ^2^_e_, are additive direct, additive maternal, maternal permanent environmental and residual variances, respectively. The total heritability (h^2^_t_), was calculated using the formula h^2^_t_ = (h^2^+ 0.5 m^2^+1.5mr_am_h) [16]. The best model suited for each trait considering the likelihood ratio test was chosen and used to study the genetic parameters [17].

For the bivariate analysis, the best models from the single trait analyses were combined with appropriate covariance between random effects in the model. The best model identified as per likelihood ratio test for specific trait was only used for the bivariate analysis with starting values derived from single trait analysis. Estimates of genetic parameters like genetic, phenotypic and environmental correlations between different economic traits were obtained by average information restricted maximum likelihood (AIREML) fitting an animal model WOMBAT [15]. To formally test the significance of additive genetic correlations, the log-likelihood for this model is compared to model in which COV_A_ = 0 is specified [18]. Significance of maternal permanent environmental correlations were also tested accordingly compared to model in which COV_C_ = 0. Significance of residual and phenotypic correlations was tested by hypothesis test to decide whether the value of the correlation coefficient is significantly different from zero [19].

Genetic trend was estimated by regression of the estimated BV averages on generation for trait under selection (SL6) and other economically important BW and production traits under study [19]. The BVs obtained from the best model suited for each trait were considered for the estimation of average BV.

## RESULTS

### Growth and production performance

The least squares means (LSMs) over the generations for juvenile BWs up to six weeks of age and SL6 are presented in Table 2. The BWs varied significantly (p≤0.01) among the generations at 0 day, two, four, and six weeks of age. The SL6 was significantly (p≤0.01) different among the generations. The overall LSM of SL6 was 77.44±0.05 mm, which was the primary trait of selection for the population. Hatch and sex also had the significant (p≤0.01) effect on the BWs and SL6 (Table 2). Significantly higher BWs (BW2 to BW6) and SL6 were recorded in cocks.

Production traits i.e., BW20, BW40, ASM, EP40, and EW40 of five generations were analyzed and the overall LSMs were presented in Table 3. All the production traits (BW20, BW40, ASM, EP40, and EW40) were significantly influenced by generation effect. The hatch effect was significant for ASM only, which was better in hatch 3 (Table 3). The BWs at 20 and 40 weeks of age, EP, and EW at 40 weeks of age had no hatch influence (Table 3). The overall LSMs for BW20 and BW40 were 2,125±4.82 and 2,816±6.67 g, respectively. The overall LSMs for ASM, EP40, and EW40 were 182.9±0.37 days, 50.90±0.37 eggs and 55.06±0.09 g, respectively.

### Variance components and genetic parameters

The estimates of (co) variance components and genetic parameters estimated using best model for BW0, BW2, BW4, BW6, and SL6 are presented in Table 4. The best model suitable for the trait was selected based on the likelihood ratio test on log likelihood (logL) values obtained from the WOMBAT from the six models employed for analysis. Model four with additive, maternal permanent environmental and residual effects was the best model for juvenile growth traits, except BW0 for which model five was the best in which maternal genetic effect was included in addition to the model 4.

The heritability estimates obtained based on the best model for BW0, BW2, BW4, BW6 and SL6 were 0.12±0.03, 0.10± 0.02, 0.17±0.02, 0.20±0.03, and 0.17±0.03, respectively. The heritability estimates were low to moderate in the magnitude. Model four was the best for ASM trait with moderate heritability of 0.158±0.050 and c^2^ of 0.09. For other production traits; BW20, BW40, EP40, and EW40, the model one was the best with additive direct and residual effects (Table 5). The heritability estimates for BW20 and BW40 were 0.16±0.03 and 0.24±0.05, respectively. The estimated heritability was 0.14±0.01 and 0.24±0.05 for EP40 and EW40, respectively. The heritability was low in magnitude for EP and moderate for EW.

Correlation coefficients between different traits are presented in Table 6. The direct additive genetic, residual and phenotypic correlations are less in magnitude between BW0 and other growth traits, while maternal permanent environmental correlations were between 0.50±0.10 and 0.79±0.07. The correlation coefficients between BWs and SL due to different components were higher and significant (p≤0.05), except for BW0 and SL6 which had non-significant positive association (Table 6). The direct additive genetic correlation between BW6 and BW20 and BW40 was 0.48±0.11 and 0.44±0.10, respectively, whereas the correlation from other components was negligible. The association between the BW20 and BW40 was significant (p≤0.05) up to 70% from direct additive effects.

ASM had significant (p≤0.05) negative association with EP40 for direct additive, residual and phenotypic correlation which was in desirable direction. The magnitude of association was very low between ASM and EW40. The association between ASM and BWs was negative with less magnitude for all the components (Table 6). The correlation between EP40 and BW40 was negative from direct additive, residual and phenotypic components, while it was positive with less magnitude between EP40 and BW20 for phenotypic correlation only. The EP and EW had negative correlation of lower magnitude. The EW and BWs had positive additive genetic correlation, while residual and phenotypic correlations were negligible.

**Figure 1:**
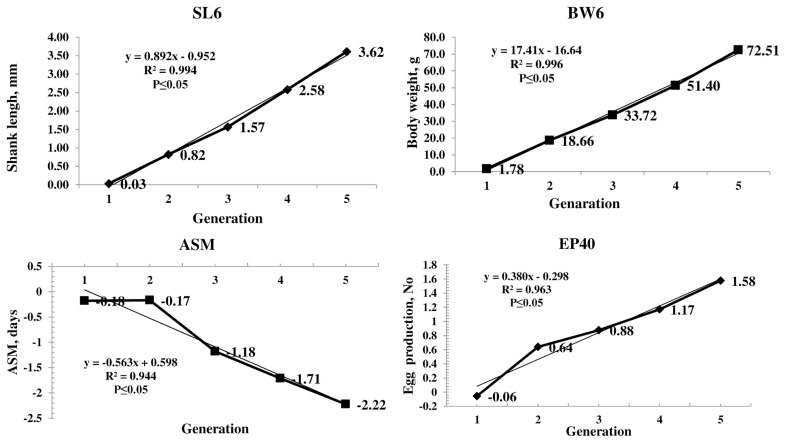
Genetic trends of average breeding values of primary (SL6) and important correlated traits (BW6, ASM, and EP40). Average breeding value of SL6, BW6, and EP40 increased significantly in a linear direction and decreased in ASM over the generations. SL6, shank length at six weeks of age; BW6, body weight at six weeks of age; ASM, age at sexual maturity; EP40, egg production up to 40 weeks of age.

**Figure 2:**
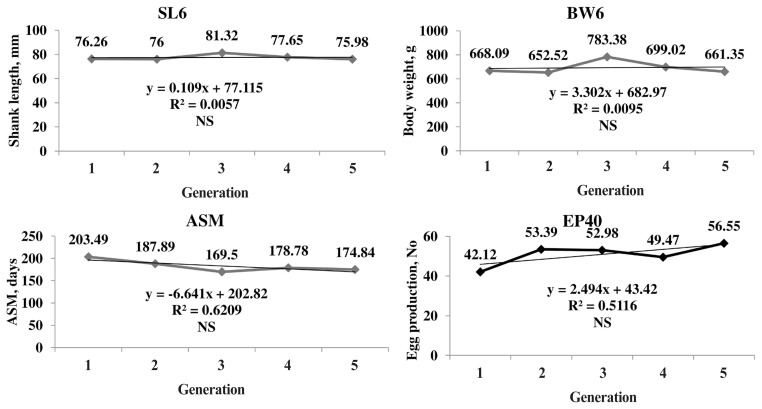
Phenotypic trends of primary (SL6) and important correlated traits (BW6, ASM, and EP40). Phenotypic trend increased for SL6, BW6, and EP40 and decreased for ASM in positive direction over the generations. SL6, shank length at six weeks of age; BW6, body weight at six weeks of age; ASM, age at sexual maturity; EP40, egg production up to 40 weeks of age; NS, non significant.

The genetic trend of the primary trait and correlated traits are presented in Figure 1. The genetic trend, estimated by regression of the estimated BV on generation was significant (p≤0.05) for SL6, the trait under selection, BW and production traits. The BV of SL6 in the population increased linearly from 0.03 to 3.62 mm due to selection during the last four generations. The average genetic gain was 0.89 mm per generation for SL6. The BV of BW6 also increased linearly from 1.78 to 72.51 g with significant (p≤0.05) genetic response of 17.42 g for each generation. The phenotypic trend of SL6, BW6, ASM, and EP40 was presented in Figure 2. The phenotypic trend of all the traits was in favourable direction with various magnitudes.

Among the production traits, ASM reduced linearly with an annual reduction of 0.56 days in each generation. The BV of EP at 40 weeks of age increased linearly. The average genetic gain for each generation was significant (p≤0.05) over the five generations with an average increase of 0.38 eggs per generation. The EW 40 also showed the linear trend with an average genetic gain of 0.12 g per generation. The average inbreeding coefficient of the population was 0.02 and that of inbred birds was 0.04 in the population.

## DISCUSSION

The importance of partitioning and attributing the variance to various non genetic factors like maternal permanent environment, residual and phenotypic effects in addition to the direct additive effects for a quantitative trait was discussed in this article. The traits which had significant effect of generation, hatch and sex were further utilized in the model for the estimation of the variance components and genetic parameters.

The LSMs for BWs significantly varied in different generations, hatches and sex in the population (Table 2). Many authors reported significant effect of hatch and sex on BW and SL [10,11,20]. The probable reason might be the variation of environmental factors (maternal effect, hatching conditions, and rolling reactions) over five generations as the performance of the first hatch was better than subsequent hatches. Due to sexual dimorphism, males grow faster than females leading to significant differences between them. The differences in generations show that selection SL6 over the generations was operating in the population in a positive direction with significant effect. Similar, positive selection response for primary traits was observed in PD-1 line and Punjab broiler-1 (PB-1) line [2,21]. However, it may not be true always as these might be influenced by many other non-genetic factors too.

The LSMs for ASM significantly and gradually reduced over the generations in desired direction, which may be due to the correlated response to the selection for SL6. The SL is positively correlated to BW and higher BW at ASM ultimately leads to reduction in ASM though direct selection was not practiced for the trait [21]. Current analysis revealed that higher the BWs at 20 weeks, lesser the ASM. It was concluded that early BW can influence the onset of EP leading to reduced ASM which was similar to the present findings [22,23]. ASM was negatively correlated with egg numbers. The differences in BW20 and BW40 might be attributable to the type of feed and feed restriction schedule followed for maintaining the BW at laying. The EP40 and EW40 showed a significant increasing trend over the generations, which might be due to the correlated response to selection trait [21].

Model four with additive, maternal permanent environ mental, residual and phenotypic effects was the best model for juvenile BW and SL, except for BW0. For BW0, the Model five was the best model, which has maternal genetic effects in addition to effects of model 4. Maternal effects are proportional to the contribution of maternal additive, dominant gene effects and the differences in allele frequencies between favorable and unfavorable alleles [8]. Maternal environment effects in chicken are pre-ovipositional and post-ovipositional. Egg quality traits like EW, size and shell quality, which were determined by the maternal inheritance influence the chick weight at hatch which was also true in the present study as the BW0 had significant maternal genetic effects [24]. As the age advanced, the maternal effects reduced as the chicks are reared under artificial brooding. One worker reported that the contribution of maternal effects to the phenotypic variation of BW decreases with age [25]. Similar findings of reduced maternal effects were reported in crosses involving broiler lines [8]. Many authors reported that maternal genetic effects were essential for early BW (hatch weight), although the contribution of maternal permanent environmental effects was more than the direct and maternal genetic effects [5,6]. It was observed that the maternal genetic effects on BWs up to 12 weeks of age in dual purpose chicken contrary to the present study [26]. The maternal genetic effect had no additional effect when the permanent environmental effect was included for the traits, BW2, BW4, BW6, and SL6. This is in agreement with earlier work where it was reported that the inclusion of one of the maternal effects in the model could be enough to adjust the variation occurring in both effects [15]. The variations in maternal effects observed in the literature may be due to the breed variations of populations utilized in the studies and the type of management adopted during the experimental period.

Model 1 with direct additive effects was the best model for all the production traits (BW20, BW40, EP40, and EW40), except for ASM, which showed the maternal effect (permanent environmental) of lesser magnitude. As the age advanced, the maternal effects gradually reduced and only the direct additive genetic effects prevailed. This may be the reason that all the production traits have only direct additive genetic effects in the present study. The direct additive effects model without or negligible maternal effects were suitable for EP, which was true in the present study also [6,27]. However, some authors reported maternal effects in addition to the direct additive effects for the EP and EW [26,28]. However, based on the magnitude of contribution and significance of the maternal effects, these may be included in the model for avoiding bias and achieving higher precision of the genetic parameters.

The heritability estimates of BWs and SL length estimated using REML were lesser as compared to the traditional Henderson variance component (full sib) analysis and the model one (direct additive) of WOMBAT animal model. The h^2^ estimated using REML animal model was more precise as it reduced the overestimation of the genetic parameters by partitioning the variance and covariance in to maximum possible components. Similar findings of less magnitude for h^2^ were reported by many authors using REML [3,5,6,26]. Maternal effects account for small part of the variability of the economic traits (2% to 8%), but ignoring them will lead to significant overestimation of the h^2^ [27,29]. The non-inclusion of maternal effects in the model, despite their existence will result in overestimation of the direct heritability and the consequent wrong conclusion, and defective breeding programs [4]. In the present study also, ignoring the maternal effects in the model resulted in overestimation of direct h^2^ from 0.17 (model four) to 0.28 (model one) for SL 6, the primary trait of selection. Similar results were observed for other traits also.

Positive genetic correlation indicates that selection for one trait can improve the performance in other traits and the negative correlation reduces the performance [2,9]. The correlation coefficients of maternal permanent environmental effects were higher and significant (p≤0.05) between BW0 and other juvenile BWs compared to other components, which clearly revealed that there was a significant maternal effect on BW0 (Table 6). It indicated that the non-genetic factors like mothering ability and uterus size have great influence on early BW, which reduces or becomes negligible later on. Therefore, selection based on early BW may not be a wise criterion for selecting individuals for higher BW. The correlation coefficient between other BWs and SL were as per expectations with high degree of positive and significant association from all the components. Similar findings of high genetic correlations were observed between BW and SL in naked neck chicken and native chickens [9,10]. The ASM and EP40 were negatively correlated with significant (p≤0.05) direct additive genetic correlations, which were observed in this study too [9]. The ASM and EW were directly associated since higher the ASM, more was the EW. ASM and BWs (BW20 and BW40) recorded negative association which was desirable as heavier birds matured earlier similar to the observation made by Rajkumar et al [9,11]. The BWs and EP had negative correlation for additive, residual and phenotypic components. It is an established fact that BW and EP are negatively correlated traits and the present findings also substantiate this [5,9,30]. The precise estimation of correlation coefficients from different components helps the breeder in multi-trait selection programs based on the significance and direction of the association. The inclusion of traits with higher and significant association may result in simultaneous improvement of the traits.

The genetic trend showed that the selection was operating with an average genetic gain of 0.89 mm in SL, which was the primary trait of selection. The average BV of the population increased significantly in a linear direction indicating the effectiveness of selection (Figure 1). Similar trend was observed in BW4 and BW6 as correlated responses as SL and BW are highly correlated traits [2]. The BW20 and BW40 also showed the significant positive linear trend as a correlated response.

The BV of ASM gradually reduced over the generations which were in desired direction. The EP 40 BV showed a linear positive trend with an average genetic gain of 0.38 eggs per generation. Similar trend was observed in EW also. The response observed in all the production traits is due to the correlated response as the traits were not included in the selection program.

The rate of inbreeding in the population was very low, which may be because of adoption of proper and effective breeding plan where care was taken that close relatives of two generations were not allowed to mate. The status of the population at the end of the 5th generation is ideal with 0.032 inbreeding coefficient. The inbreeding was negligible till this generation, however, may increase in further generations due to the selection.

The study concluded that the population is in ideal condition without any deleterious effects of inbreeding and the selection is quite effective with significant genetic gains in each generation for primary trait of selection and other associated traits. The fact that precise estimation of genetic parameters, heritability and correlation with REML model further improve accuracy of the BV estimates, thus enabling the breeder’s decision making related to the selection and breeding strategy more accurate and ultimately aiding in genetic improvement of the populations.

## Figures and Tables

**Table 1:** Characteristics of data on juvenile and production traits of PD-1 line

Particulars	BW01)	BW21)	BW41)	BW61)	SL61)	BW201)	BW401)	ASM1)	EP401)	EW401)
No. of records	14,372	13,579	13,299	13,331	13,315	1,998	1,782	1,835	1,616	1,574
No. of sires	249	249	249	249	249	245	244	244	240	243
No. of sires with records and progeny in data	199	199	198	199	199	-	-	-	-	-
No. of dams	1,086	1,081	1,077	1,076	1,077	730	697	691	668	659
No. of dams with records and progeny in data	854	854	850	851	852	577	532	555	502	472
Mean	37.84 g	135.83 g	355.42 g	692.88 g	77.43 mm	2,124.35 g	2,827.34 g	182.96 d	51.38 no	55.04 g
SD	3.47	29.80	81.94	128.08	3.21	248.70	292.37	19.29	15.06	3.86

SD, standard deviation; PD-1, Vanaraja male line.

1) BW0, day old body weight; BW2, 2nd week body weight; BW4, 4th week body weight; BW6, 6th week body weight; SL6, 6th week shank length; BW20, 20th week body weight; BW40, 40th week body weight; ASM, age at sexual maturity; EP40, 40 week egg production; EW40, 40 week egg weight.

**Table 2:** Least squares means (mean±SE) of juvenile growth traits of PD-1 line

Particulars	Body weight at different weeks (g)				Shank ength (mm)
	
	0 d	2nd	4th	6th	6th
Overall LSM	37.78±0.03 (14,379)	135.47±0.22 (13,586)	354.88±0.62 (13,306)	692.87±1.00 (13,338)	77.44±0.05 (13,322)
Generation	**	**	**	**	**
1	37.69±0.06^ab^ (3,102)	133.69±0.45^c^ (3,079)	345.66±1.26^b^ (3,052)	668.09±2.04^c^ (3,094)	76.26±0.10^a^ (3,102)
2	37.79±0.07^ab^ (2,486)	118.07±0.54^a^ (2,460)	313.44±1.47^a^ (2,466)	652.52±2.39^a^ (2,484)	76.00±0.12^a^ (2,486)
3	38.18±0.07^c^ (2,678)	161.23±0.49^e^ (2,678)	433.54±1.35^d^ (2,677)	783.38±2.21^e^ (2,677)	81.32±0.11^c^ (2,674)
4	37.66±0.06^ab^ (3,088)	143.45±0.47^d^ (2,958)	364.09±1.28^c^ (2,928)	699.02±2.09^d^ (2,938)	77.65±0.10^b^ (2,939)
5	37.57±0.06^a^ (3,025)	120.90±0.53^b^ (2,411)	317.64±1.52^a^ (2,187)	661.35±2.49^b^ (2,145)	75.98±0.12^a^ (2,121)
Hatch	**	**	**	**	**
1	36.75±0.06^a^ (3,554)	143.50±0.43^d^ (3,515)	367.11±1.20^a^ (3,508)	732.05±1.96^c^ (3,517)	79.17±0.09^d^ (3,518)
2	38.01±0.05^c^ (3,929)	135.46±0.41^b^ (3,836)	341.89±1.13^b^ (3,771)	671.85±1.85^a^ (3,804)	76.72±0.09^b^ (3,813)
3	37.51±0.06^b^ (2,660)	137.92±0.51^c^ (2,528)	368.40±1.10^a^ (2,454)	694.87±2.31^b^ (2,469)	77.71±0.11^c^ (2,474)
4	38.82±0.06^d^ (4,236)	125.00±0.43^a^ (3,707)	342.09±1.21^b^ (3,573)	672.71±1.98^a^ (3,548)	76.17±0.09^a^ (3,517)
Sex	NS	**	**	**	**
Male	37.87±0.04 (6,551)	138.53±0.37^b^ (6,484)	363.43±1.01^b^ (6,493)	724.52±1.45^b^ (6,558)	78.97±0.07^b^ (6,552)
Female	37.76±0.04 (6,787)	135.01±0.36^a^ (6,691)	348.57±0.99^a^ (6,685)	661.22±1.39^a^ (6,780)	75.91±0.07^a^ (6,770)

SE, standard error; PD-1, Vanaraja male line; LSM, least squares mean; NS, non significant.

Values in the parentheses are number of observations;

** (p<0.01).

a–e Means with different superscripts in the same column within the same parameter differ significantly at p<0.01.

**Table 3:** Least squares means (mean±SE) of production traits of PD-1 line

Particulars	ASM1) (d)	BW201) (g)	BW401) (g)	EP401) (no)	EW401) (g)
Overall LSM	182.90±0.37 (1,834)	2,124.92±4.82 (1,998)	2,815.77±6.67 (1,782)	50.90±0.37 (1,616)	55.06±0.09 (1,574)
Generation	**	**	**	**	**
1	203.49±0.83^e^ (343)	1,899.44±10.76^a^ (388)	2,657.53±17.52^a^ (253)	42.12±0.91^d^ (253)	54.92±0.23^bc^ (283)
2	187.89±0.79^d^ (437)	2,075.05±10.35^b^ (468)	2,732.59±14.12^b^ (423)	53.39±0.73^b^ (395)	54.53±0.23^c^ (309)
3	169.50±0.78^a^ (405)	2,291.66±10.65^e^ (405)	2,947.46±13.66^d^ (405)	52.98±0.76^b^ (368)	55.31±0.19^b^ (405)
4	178.78±0.86^c^ (324)	2,197.92±11.77^d^ (324)	2,807.35±15.03^c^ (324)	49.47±0.85^c^ (286)	54.35±0.21^c^ (324)
5	174.84±0.88^b^ (329)	2,175.54±10.72^c^ (413)	2,933.95±14.33^d^ (377)	56.55±0.83^c^ (314)	56.18±0.24^a^ (253)
Hatch	**	NS	NS	NS	NS
1	182.63±0.70^ab^ (529)	2,145.79±8.92 (591)	2,803.82±11.45 (587)	49.49±0.67 (496)	54.98±0.18 (493)
2	183.83±0.70^ab^ (491)	2,121.58±9.40 (519)	2,835.05±12.37 (485)	51.71±0.71 (421)	55.21±0.09 (398)
3	180.46±0.84^a^ (346)	2,110.03±10.97 (379)	2,797.34±15.12 (323)	50.93±0.84 (297)	54.81±0.22 (304)
4	184.69±0.75^b^ (468)	2,122.28±9.86 (509)	2,826.89±15.19 (387)	51.38±0.76 (402)	55.24±0.21 (379)

SE, standard error; PD-1, Vanaraja male line; LSM, least squares mean; NS, non significant.

1) ASM, age at sexual maturity; BW20, 20th week body weight; BW40, 40th week body weight; EP40, 40 week egg production; EW40, 40 week egg weight.

Values in the parentheses are number of observations;

** (p<0.01).

a–e Means with different superscripts in the same column within the same parameter differ significantly at p<0.01.

**Table 4:** Estimates of (co)variance components and genetic parameters for juvenile traits in PD-1 line

Components2)	BW01)	BW21)	BW41)	BW61)	SL61)

	Model 5	Model 4	Model 4	Model 4	Model 4
σ^2^_a_	1.49±0.32	65.50±12.87	823.9±125.36	2,621.53±360.09	5.371±0.840
σ^2^_m_	3.36±0.59	-	-	-	-
σ^a^_m_	-	-	-	-	-
σ^2^_c_	2.32±0.40	37.86±5.37	179.87±38.00	397.41±96.36	1.144±0.25
σ^2^_e_	5.75±0.18	539.39±9.59	3,940.8±82.76	10,179.7±228.96	25.742±0.55
σ^2^_p_	12.92±0.37	642.76±8.97	4,944.6±74.88	13,198.7±207.60	32.26±0.49
h^2^	0.12±0.03	0.10±0.02	0.17±0.02	0.20±0.03	0.17±0.03
m^2^	0.26±0.04	-	-	-	-
r_am_	-	-	-	-	-
c^2^	0.18±0.03	0.06±0.01	0.04±0.01	0.03±0.01	0.035±0.01
h^2^_T_	0.25	0.10	0.17	0.20	0.17
logL	−21,875.14	−50,307.56	−62,737.59	−69,356.47	−29,336.41

Values after ± are standard errors.

PD-1, Vanaraja male line.

1) BW0, day old body weight; BW2, 2nd week body weight; BW4, 4th week body weight; BW6, 6th week body weight; SL6, 6th week shank length.

2) σ^2^_a_, σ^2^_c_, σ^2^_m_, σ^2^_e_, and σ^2^_p_ are additive direct, maternal permanent environmental, maternal genetic, residual variance and phenotypic variance, respectively; h^2^ is heritability; c^2^ is σ^2^_c_/σ^2^_p_; h^2^_T_ is total heritability and log L is log likelihood for the model obtained from WOMBAT.

**Table 5:** Estimates of (co)variance components and genetic parameters for production traits PD-1 line

Components2)	ASM1)	BW201)	BW401)	EP401)	EW401)

	Model 4	Model 1	Model 1	Model 1	Model 1
σ^2^_a_	38.22±12.47	7,009.29±1,798.80	17,923.6±3,807.34	29.940±8.66	3.512±0.82
σ^2^_m_	-	-	-	-	-
σ^a^_m_	-	-	-	-	-
σ^2^_c_	23.23±7.69	-	-	-	-
σ^2^_e_	180.67±10.25	3,8217.7±1,812.2	56,607.8±3,283.27	177.78±9.13	11.343±0.71
σ^2^_p_	242.12±8.68	45,227.0±1,497.7	74,531.4±2,724.18	207.75±7.58	14.86±0.57
h^2^	0.16±0.05	0.16±0.04	0.24±0.05	0.14±0.01	0.24±0.05
m^2^	-	-	-	-	-
r_am_	-	-	-	-	-
c^2^	0.10±0.03	-	-	-	-
h^2^_T_	0.16	0.16	0.24	0.14	0.24
logL	−5,903.19	−11,666.73	−10,826.46	−5,099.69	−2,879.23

Values after ± are standard errors.

1) ASM, age at sexual maturity; BW20, 20th week body weight; BW40, 40th week body weight; EP40, 40 week egg production; 40EW, 40 week egg weight.

2) σ^2^_a_, σ^2^_c_, σ^2^_m_, σ^2^_e_, and σ^2^_p_ are additive direct, maternal permanent environmental, maternal genetic, residual variance and phenotypic variance, respectively; h^2^ is heritability; c^2^ is σ^2^_c_/σ^2^_p_; h^2^_T_ is total heritability and log L is log likelihood for the model obtained from WOMBAT.

**Table 6:** Correlations coefficients (r±SE) between different juvenile and production traits

Trait combinations1)	Direct additive genetic correlation (r_a_)	Maternal permanent environmental correlations (r_c_)	Residual effect correlations (r_e_)	Phenotypic correlations (r_p_)
Juvenile traits				
BW0 and BW2	0.31±0.14*	0.79±0.07*	0.12±0.02*	0.20±0.01*
BW0 and BW4	0.07±0.13^NS^	0.64±0.10*	0.12±0.02*	0.14±0.01*
BW0 and BW6	−0.08±0.12^NS^	0.74±0.11*	0.12±0.02*	0.118±0.01*
BW0 and SL6	0.05±0.13^NS^	0.495±0.10*	0.090±0.02*	0.102±0.01*
BW2 and BW4	0.93±0.03*	0.75±0.06*	0.624±0.01*	0.67±0.01*
BW2 and BW6	0.82±0.05*	0.74±0.08*	0.420±0.01*	0.53±0.01*
BW2 and SL6	0.71±0.06*	0.75±0.07*	0.50±0.01*	0.53±0.01*
BW4 and BW6	0.97±0.01*	0.833±0.05*	0.693±0.01*	0.749±0.01*
BW4 and SL6	0.81±0.04*	0.917±0.05*	0.695±0.01*	0.723±0.01*
BW6 and SL6	0.98±0.04*	0.909±0.04*	0.789±0.01*	0.794±0.01*
BW6 and BW20	0.48±0.11*	-	0.06±0.03*	0.14±0.03*
BW6 and BW40	0.44±0.10*	-	0.08±0.04*	0.16±0.03*
BW20 and BW40	0.72±0.10*	-	0.38±0.03*	0.45±0.02*
Production traits				
ASM and EP40	−0.68±0.15*	-	0.38±0.03	−0.41±0.02*
ASM and EW40	0.22±0.19 ^NS^	-	0.10±0.04*	0.12±0.03*
ASM and BW20	−0.32±0.18*	-	0.33±0.04*	−0.31±0.02*
ASM and BW40	−0.10±0.18^NS^	-	−0.03±0.04^NS^	−0.04±0.03^NS^
EP40 and BW20	0.18±0.19^NS^	-	0.18±0.04*	0.13±0.03*
EP40 and BW40	−0.31±0.16*	-	0.13±0.04*	−0.16±0.03*
EP40 and EW 40	−0.31±0.17^NS^	-	−0.03±0.04^NS^	−0.08±0.03*
EW40 and BW20	0.33±0.16^NS^	-	−0.01±0.04^NS^	0.06±0.03*
EW40 and BW40	0.41±0.14*	-	0.07±0.04*	0.15±0.03*

SE, standard error; NS, non significant.

1) BW0, day old body weight; BW2, 2nd week body weight; BW4, 4th week body weight; BW6, 6th week body weight; SL6, 6th week shank length; BW20, 20th week body weight; BW40, 40th week body weight; ASM, age at sexual maturity; EP40, 40 week egg production; EW40, 40 week egg weight.

* p≤0.05; correlation coefficients with * superscript is significant at p≤0.05.
